# Endogenous *Fusarium* Endophthalmitis after Bone Marrow Transplant: A Case Report and Literature Review

**DOI:** 10.3390/vision8030044

**Published:** 2024-07-21

**Authors:** Cindy S. Zhao, Karen Wai, Eubee B. Koo, Ehsan Rahimy, Prithvi Mruthyunjaya, Vinit B. Mahajan, Charles M. T. DeBoer

**Affiliations:** 1 Department of Ophthalmology, Byers Eye Institute, Stanford University, Palo Alto, CA 94303, USA; 2 Department of Ophthalmology, Palo Alto Medical Foundation, Palo Alto, CA 94301, USA; 3 Molecular Surgery Laboratory, Stanford University, Palo Alto, CA 94303, USA; 4 Veterans Affairs Palo Alto Health Care System, Palo Alto, CA 94304, USA

**Keywords:** endophthalmitis, fungemia, *Fusarium*, antifungal

## Abstract

Purpose: We aim to present a case of disseminated fusariosis that occurred in the setting of immunosuppression and presented with bilateral endogenous endophthalmitis, along with a literature review of *Fusarium* endophthalmitis, highlighting management strategies. Observation: A 70-year-old male with acute myeloid leukemia who had recently undergone a bone marrow transplant noted bilateral floaters and decreased vision. He was found to have bilateral *Fusarium* endophthalmitis, with subsequent evidence of fungemia and fusariosis in his skin and joints. Despite aggressive local and systemic treatment, he succumbed to the disease. Endophthalmitis was initially stabilized with pars plana vitrectomy and intravitreal amphotericin and voriconazole until the patient transitioned to comfort measures. A review of 31 cases demonstrates that outcomes are poor and that the disease must be treated aggressively, often both systemically and surgically. Conclusion: This case highlights the recalcitrance of *Fusarium* bacteremia and *Fusarium* endophthalmitis.

## 1. Introduction

Although ubiquitous in the environment as a plant pathogen, *Fusarium* does not commonly result in systemic fungal infection or enter the eye to cause endophthalmitis [1]. When present, *Fusarium* endophthalmitis more commonly occurs exogenously after inoculation from trauma, invasion into the eye from keratitis, or after cataract surgery. Often, in these cases, aggressive treatment is required, and prognosis remains poor despite multifaceted local, surgical, and systemic treatment [2,3,4,5]. This report illustrates a case of bilateral endogenous *Fusarium* endophthalmitis in an immunocompromised patient, highlighting the complex multidisciplinary care these rare cases require.

## 2. Case Report

A 70-year-old male with acute myeloid leukemia (AML) presented for a mismatched, unrelated donor bone marrow transplant (BMT). He had previously been treated with three cycles of chemotherapy, including two rounds of intrathecal chemotherapy for presumed central nervous system disease. While the central nervous system disease showed improvement, the patient had residual bone marrow involvement.

Despite a hospital course complicated by neutropenic fever, acute pulmonary edema, new-onset atrial fibrillation, and clostridium difficile colonization, his bone marrow transplant team felt optimistic one month after his BMT that his immune system was recovering and that his disease may have been cured. Throughout this first month after BMT, he was on antibacterial, antiviral, and antifungal agents for prophylaxis: oral levofloxacin for neutropenic fever, oral acyclovir for herpes simplex virus (HSV) and varicella-zoster virus (VZV), oral letermovir for cytomegalovirus (CMV), oral trimethoprim/sulfamethoxazole for pneumocystis jiroveci pneumonia, and oral isovuconazole for fungal prophylaxis.

**Figure 1:**
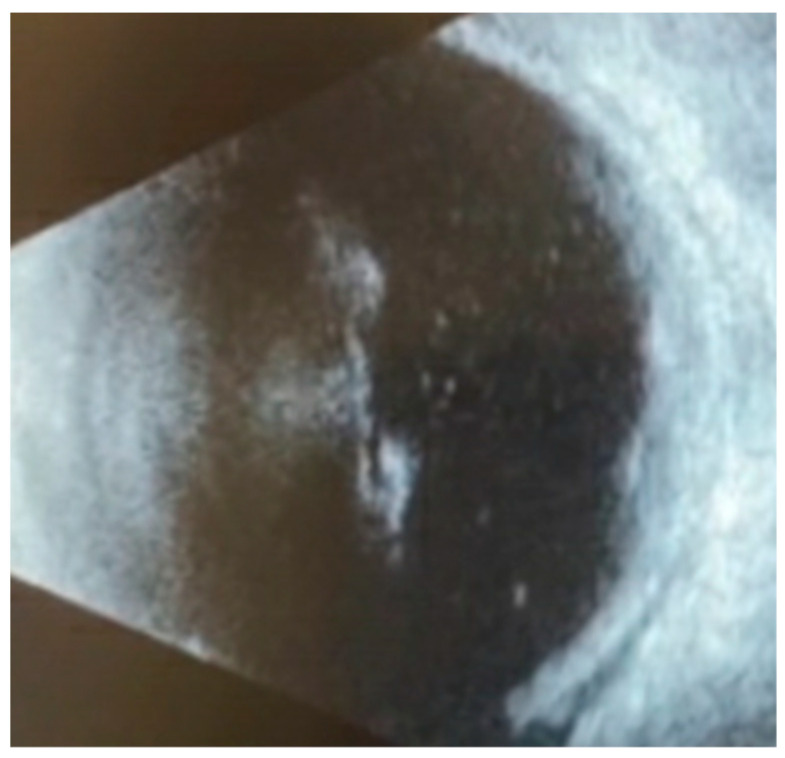
B-scan ultrasonography of macular lesions in the left eye on initial examination demonstrated vitreous opacities consistent with vitritis, elevated subretinal lesions, and a thickened choroid.

It was at this time, one month following BMT, that the patient noted one week of bilateral floaters and decreased vision. An ophthalmology consult was requested. On exam, his visual acuity was counting fingers from two feet in both eyes with normal intraocular pressures. He had mild temporal conjunctival injection in the left eye but an otherwise normal anterior segment exam. The dilated fundus exam was notable for mild vitreous haze bilaterally, numerous foci of elevated, subretinal lesions, and overlying hemorrhage in the macula of each eye. Ocular ultrasonography (Figure 1) confirmed the presence of subretinal masses and vitreous debris bilaterally, without retinal detachment.

**Figure 2:**
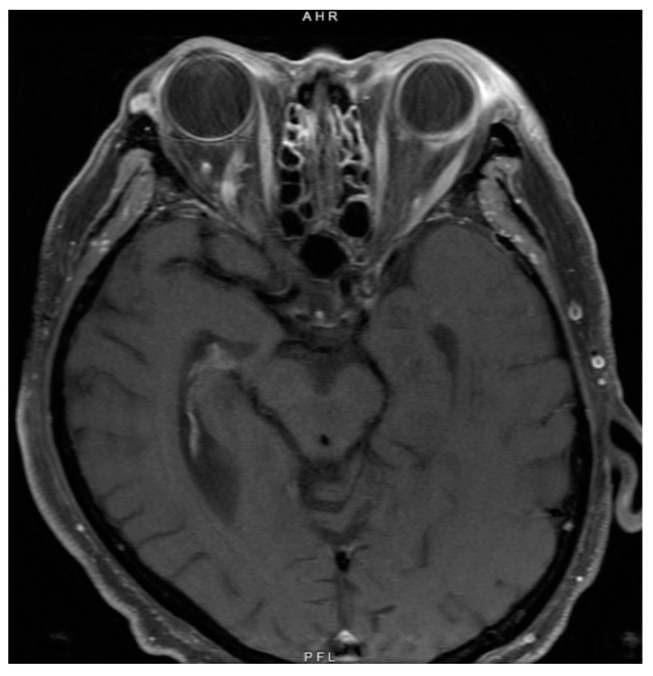
Magnetic resonance imaging of the brain and orbit at the time of initial diagnosis showed abnormal thickening of the left sclera and periorbital soft tissues but no lesions infiltrating the optic nerve to suggest malignancy.

**Figure 3:**
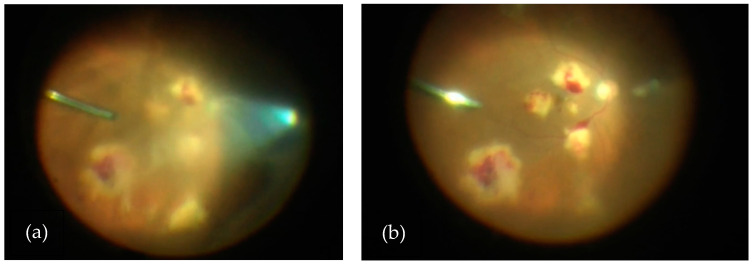
(**a**) View of vitritis and subretinal lesions in the right eye during diagnostic vitrectomy and prior to core vitrectomy. (**b**) View of subretinal lesions in the right eye during diagnostic vitrectomy and after core vitrectomy.

**Figure 4:**
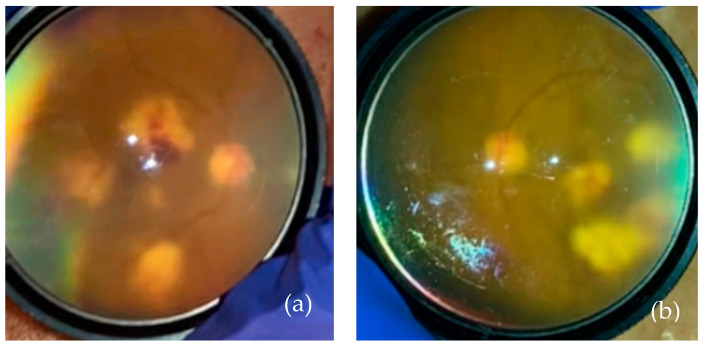
(**a**) Right eye post-operative day 2 after diagnostic vitrectomy of the right eye. (**b**) The left eye on the same day.

Due to concern for recurrent or widespread malignancy, magnetic resonance imaging (Figure 2) and a lumbar puncture were performed, neither of which showed evidence of neoplasia. The patient received a vitreous tap and intravitreal injection of voriconazole (100 mg/0.1 mL), vancomycin (1 mg/0.1 mL), and ceftazidime (2.25 mg/0.1 mL) in both eyes. After five days, the culture began to grow fungi, which ultimately speciated as *Fusarium solani*. A blood culture and diagnostic vitrectomy (Figure 3) similarly yielded *Fusarium* species. His vision remained intact, able to count fingers from two feet in both eyes afterward (Figure 4). Further examination revealed systemic dissemination with the involvement of his skin and several joints, which similarly grew *Fusarium* species. From the blood sample, amphotericin B was found to have a minimum inhibitory concentration (MIC) of 2 micrograms/mL and voriconazole an MIC of 4 mg/mL.

Systemic coverage was initiated with intravenous amphotericin B and voriconazole and, eventually, a novel experimental antifungal, fosmanogepix. Ocular treatment consisted of ten rounds of intravitreal injections every other day with antifungals, alternating between amphotericin B and voriconazole. Examination findings stabilized during treatment, with similar visual acuity and no extension of the lesions. Given extensive systemic disease, the patient ultimately wished to discontinue local treatments and withdrew care, succumbing to his disease process.

## 3. Discussion

Even with aggressive systemic and local therapy, fungal endophthalmitis can be a devastating disease with a poor visual prognosis. Fungal endophthalmitis more often occurs exogenously after trauma, keratitis, or intraocular surgery; endogenous endophthalmitis associated with systemic fungal infection represent 2–15% of all cases [6]. The most common organisms found in endogenous fungal endophthalmitis are *Candida albicans* and *Aspergillus* species [7]. Here, we present a case of bilateral endogenous fungal endophthalmitis caused by *Fusarium solani*, which is a rare presentation with isolated case reports in the literature, as summarized below.

## 4. Literature Review

Twelve species of *Fusarium* have been reported to be associated with systemic infection, of which the most common are *Fusarium solani* (~50% of cases), *Fusarium oxysporum* (~20% of cases), *Fusarium verticillioidis* (~10% of cases) and *Fusarium monoliforme* (~10% of cases) [1]. When associated with ocular infection, *Fusarium* species are more often associated with keratitis and exogenous endophthalmitis [1,6].

A literature review was conducted on April 10, 2024, on PubMed using the keywords “fusarium endophthalmitis” and “endogenous fusarium endophthalmitis.” All cases of endogenous *Fusarium* endophthalmitis referred to in these articles were also included. Several larger case series reported exogenous *Fusarium* endophthalmitis, while a limited number of case reports associated with endogenous *Fusarium* endophthalmitis were found (*n* = 30, not including the case reported here). Prior to this review, the largest case series included 14 cases [8], so this is the most comprehensive literature review to our knowledge to date. The results are summarized in Table 1.

Most endogenous *Fusarium* endophthalmitis occurred in the setting of an immunocompromised state. Including our patient, AML (*n* = 13/31) and acute lymphocytic leukemia (*n* = 8/31) were the most commonly associated medical comorbidities. Other identified medical risk factors included immunosuppression from Hodgkin lymphoma (*n* = 1/31) [9], the use of systemic steroids (*n* = 3/31) [8,10,11], diabetes (*n* = 2/31) [8,10], Acquired immunodeficiency syndrome (AIDS, *n* = 1/31) [12], and a history of liver transplantation (*n* = 1/31) [13]. Aside from the presented case, one other patient (*n* = 2/31) had undergone a bone marrow transplant [14], while one had undergone a hematopoietic stem cell transplant (*n* = 1/31) [15]. One case for each was reported where the only identifiable medical history was intravenous drug use [16], preceding kidney infection [17], or a recent thorn prick with a self-limited local inflammatory reaction [18]. When a species was identified, *Fusarium solani* (*n* = 17/31) predominated. Two cases were reported for *Fusarium dimerum* [16,19].

Treatment options for fungal endophthalmitis include topical natamycin (though not often used alone), intravitreal injections of antifungals such as amphotericin B (5–10 µg/0.1 mL) and voriconazole (1 mg/0.1 mL), systemic oral or intravenous antifungal treatment with amphotericin B, and surgical treatment with pars plana vitrectomy (*n* = 17/31) or the removal of other infected areas (e.g., lensectomy) (*n* = 4/31) and in recalcitrant cases, evisceration or enucleation (*n* = 7/31). Almost all authors used systemic amphotericin (*n* = 24/31) and/or an azole (*n* = 14/31) such as voriconazole (most common, *n* = 12), fluconazole (*n* = 1), or itraconazole (*n* = 1). Intravitreal injections of amphotericin only (*n* = 10/31), vorizonacole only (*n* = 6/31), or an alternating combination of amphotericin and voriconazole (*n* = 6/31) were given at most, simultaneously and once weekly, up to 6 weeks [20] (aside from the reported case)**.** Seven cases received systemic therapy without intravitreal injections, with one of these patients undergoing pars plana vitrectomy and one requiring enucleation. No well-studied regimen exists to guide treatment selection, and most cases used combination therapy. Vitrectomy provides the advantage of reducing the fungal burden in the vitreous and provides additional samples for establishing the diagnosis, as well as addressing common post-infectious sequelae such as retinal detachment and epiretinal membrane [21]. In one case of fungal endophthalmitis (not specified to be *Fusarium*), the intralesional injection of voriconazole and povidone-iodine was performed with final vision at 20/25 [22].

Systemic outcome and final vision varied without a consistent pattern. Two cases had patients that ultimately recovered to 20/20 visual acuity; both of these cases involved only the eye and suggested the importance of immune recovery and treatment with vitrectomy, systemic amphotericin B, and intravitreal injections of antifungals [16,20]. Overall, this literature review suggests the need for aggressive treatment and a surgical reduction in disease burden, but no conclusion can be drawn about the most effective treatment nor about systemic and ocular prognosis upon diagnosis. Only three patients had final visual acuities between 20/50 and 20/80, while most patients experienced near or total vision loss of the affected eyes [8,17,23]. Multi-system *Fusarium* involvement (*n* = 21/31) resulted in both survival (*n* = 11 of 24 with reported systemic outcomes) and death (*n* = 13 of 24 with reported systemic outcomes), likely in part related to factors involved in the course of their systemic disease.

**Table 1:** Summary of case reports in the literature.

Reference	No. of Patients	Age (Years)	Gender	Medical History	Antifungals Prior to Ocular Symptoms	Involved Sites	*Fusarium* species	Treatment	Ocular Surgery	Ocular Outcome	Final Vision	Systemic Outcome
Tiribelli et al. 2002 [24]	1	31	Male	AML	Itraconazole	Skin, lungs, eye, meninges	*F. solani*	Systemic: AmB	Vitrectomy Iridectomy PK	No response	Total vision loss	Death from hemorrhagic shock
								Intraocular: AmB				
Rezai et al. 2005 [25]	1	70	Female	AML	NA	Skin, blood, eye	NA	Systemic: AmB	None	No response	-	Death from multiorgan failure
								Intraocular: AmB				
Cudillo et al. 2006 [26]	1	34	Male	ALL	Itraconazole	Skin, blood, lungs, sinuses, eye	NA	Systemic: AmB	None	Response	-	Death from leukemia relapse
Thachil et al. 2010 [15]	1	67	Female	AML	NA	Skin, blood, eye	*F. solani*	Systemic: Vori, AmB	Vitrectomy	Response	HM	Survival
								Intraocular: AmB				
Kapp et al. 2011 [27]	1	69	Male	AML, HSCT	Posaconazole	Blood, kidneys, eye	*F. solani*	Systemic: Vori, AmB	Vitrectomy	No response	Worse than HM	Death from multiorgan failure
								Intraocular: AmB				
Kah et al. 2011 [23]	1	9	Male	ALL	NA	Skin, blood, eye, kidney abscess, subcutaneous soft tissue	NA	Systemic: AmB cholesterol dispersion	None	Response	VA 6/24 (20/80)	Survival
								Intraocular: Vori				
Perini et al. 2013 [28]	1	68	Male	AML	NA	Skin, blood, lungs, eye	NA	Systemic: Vori, AmB	Enucleation	No response	-	-
								Intraocular: Vori				
Malavade et al. 2013 [9]	3	62	Male	Hodgkin lymphoma	Fluconazole	Skin, eye	NA	Systemic: itraconazole, AmB	Vitrectomy	No response	-	Death from refractory leukemia
								Intraocular: AmB				
								Topical: AmB				
		66	Male	AML	AmB + Vori	Skin, bone, eye	NA	Systemic: AmB, Vori	Vitrectomy	No response	-	-
								Intraocular: AmB, Vori	RD repair Enucleation			
		46	Male	ALL	Vori	Skin, sinus, eye	NA	Systemic: Vori, AmB, co-trimoxazole	None	NA	-	-
Jørgensen et al. 2014 [13]	1	56	Female	Liver transplant x 2	Micafungin	Eye	*F. solani*	Systemic: Micafungin, Vori after enucleation	Enucleation	NA	-	Death from septic shock
								Intraocular: Vori for contralateral eye				
Bui & Carvounis 2016 [20]	1	14	Female	ALL	NA	Eye	NA	Systemic: Vori, AmB	Vitrectomy	Response	VA 20/20	Survival
								Intraocular: AmB, Vori				
Ocampo-Garza et al. 2016 [29]	1	46	Male	ALL	NA	Skin, lungs, eye	*F. solani*	Systemic: Vori, AmB	None	No response	Total vision loss	Survival, undergoing continued chemotherapy
								Topical: AmB				
Balamurugan and Khodifad 2016 [10]	1	46	Male	Diabetes mellitus, topical and oral steroids for uveitis treatment	NA	Eye	NA	Systemic: antifungal not specified	Vitrectomy	No response	HM	-
								Intraocular: Vori				
Rizzello et al. 2018 [8]	1	59	Female	ALL, Diabetes mellitus in setting of prolonged steroid use	Posaconazole	Skin, blood, eye	*F. solani*	Systemic: Vori, AmB	Vitrectomy Lensectomy	Response	VA +0.50 logMAR (20/63)	Survival in ALL remission
								Intraocular: Vori				
								Topical: AmB				
Glasgow et al. 1996 [12]	1	51	Male	AIDS, cytomegalovirus retinitis	Ganciclovir	Blood, brain, lung, kidney, thyroid, lymph nodes, eye	*F. solani*	Systemic: AmB, fluconazole	None	No response	-	Death
								Intraocular: AmB				
Patel et al. 1994 [30]	1	31	Female	ALL	NA	Skin, eye	NA	Systemic: AmB, 5-FC	None	No response	Progressive vision loss	Death from bronchopneumonia and multiorgan failure
								Intraocular: AmB				
								Topical: AmB				
Cho et al. 1973 [31]	1	2.5	Male	ALL	None	Skin, knee, eye	*F. solani*	Systemic: AmB	None	Response	-	Death
								Topical: AmB				
Simon et al. 2018 [19]	1	71	Female	AML	Posaconazole	Eye	*F. dimerum*	Systemic: Vori	Vitrectomy	Response	HM	Survival, undergoing continued chemotherapy
								Intraocular: AmB, Vori				
								Topical: Vori				
Milligan et al. 2016 [18]	1	32	Female	None; recent thorn prick with self-limited local inflammatory	None	Eye	*F. solani*	Systemic: Vori	Vitrectomy	No response	CF	Survival
								Intraocular: AmB, Vori	RD repair with silicone oil			
Yoshida et al. 2018 [32]	1	16	Male	AML	NA	Lung, spleen, subcutaneous soft tissue, eye	*F. solani*	Systemic: Vori, AmB	Vitrectomy	Response	VA 20/400	Survival
								Intraocular: Vori	Tractional RD repair			
Gabriele and Hutchins. 1996 [16]	1	30	Male	Intravenous drug use	NA	Eye	*F. dimerum*	Systemic: AmB	Vitrectomy	Response	VA 20/20	Survival
								Intraocular: AmB				
Baysal et al. 2018 [33]	1	28	Male	AML	Posaconazole	Eye	NA	Systemic: Vori, AmB	Enucleation	NA	-	Death from pneumosepsis
					Voriconazole							
Relimpio-López et al. 2018 [34]	2	NA	NA	NA	NA	Eye	*F. solani*	NA	Lensectomy	No response	-	NA
									PK			
									Evisceration			
		NA	NA	NA	NA	Eye	*F. solani*	Systemic: Vori	Vitrectomy	Response	HM (aphakic)	NA
								Intraocular: AmB, Vori	PK			
								Topical: AmB, fortified Vori, Povidone-Iodine				
Lieberman et al. 1979 [11]	1	45	Female	Uveitis treated with oral prednisone 60–120 mg daily	NA	Eye	*F. solani*	None	Sector iridectomy Lensectomy Enucleation	No response	-	NA
Comhaire-Poutchinian et al. 1990 [17]	1	27	Female	Recent kidney infection	Itraconazole	Kidney, skin, eye	NA	Systemic: AmB, 5-FC	Vitrectomy	Response	VA 4/10 (20/50)	Survival
Venditti et al. 1988 [35]	1	55	Male	AML	NA	Skin, blood, eye	*F. solani*	Systemic: AmB, 5-FC	None	No response	Total vision loss	Death
Robertson et al. 1991 [14]	1	33	Male	AML, BMT	NA	Skin, eye, toe	*F. solani*	Systemic: AmB	Vitrectomy	No response	-	Survival free of leukemia and fungal disease
								Intraocular: AmB	Enucleation			
Louie et al. 1994 [36]	1	67	Male	AML	Fluconazole	Blood, eye	*F. solani*	Systemic: AmB	Vitrectomy	Response	VA 4/200 (20/1000)	Death from recurrent leukemia
								Intraocular: AmB				
								Topical: None				
Present Case	1	70	Male	AML, BMT	isovuconazole	Skin, blood, joints, eye	*F. solani*	Systemic: AmB	Vitrectomy	No response	CF at 2-3′	Death from complications of treatment
								Intraocular: AmB, Vori				
TOTAL	31											

Abbreviations: AML, acute myeloid leukemia; AmB, Amphotericin B; 5-FC, 5-fluorocytosine; ALL, acute lymphocytic leukemia; BMT, bone marrow transplant; HSCT, hematopoietic stem cell transplant; HL, Hodgkin lymphoma; AIDS, Acquired immunodeficiency syndrome; AmB, Amphotericin B; Vori, voriconazole; NA, not available/applicable; RD, retinal detachment; PK, penetrating keratoplasty.

## 5. Conclusions

Endogenous *Fusarium* endophthalmitis is rare. When present, it is more likely to occur in the setting of an immunocompromised patient with hematologic malignancy or neutropenia. Aggressive treatment is warranted, and even then, the prognosis for visual recovery remains poor.

## Data Availability

No new data were created or analyzed in this study. Data sharing is not applicable to this article.
